# ATX-LPA_1_ axis contributes to proliferation of chondrocytes by regulating fibronectin assembly leading to proper cartilage formation

**DOI:** 10.1038/srep23433

**Published:** 2016-03-23

**Authors:** Tatsuji Nishioka, Naoaki Arima, Kuniyuki Kano, Kotaro Hama, Eriko Itai, Hiroshi Yukiura, Ryoji Kise, Asuka Inoue, Seok-Hyung Kim, Lilianna Solnica-Krezel, Wouter H. Moolenaar, Jerold Chun, Junken Aoki

**Affiliations:** 1 Graduate School of Pharmaceutical Sciences, Tohoku University, 6-3, Aoba, Aramaki-aza, Aoba-ku, Sendai, 980-8578, Japan; 2 Japan Science and Technology Agency, Precursory Research for Embryonic Science and Technology (PRESTO), Kawaguchi City, Saitama 332-0012, Japan; 3 Department of Medicine, Medical University of South Carolina, Charleston, SC 29425, USA; 4 Department of Developmental Biology, Washington University School of Medicine, St. Louis, MO 63110, USA; 5 Division of Cell Biology, The Netherlands Cancer Institute, Plesmanlaan 121, 1066CX Amsterdam, The Netherlands; 6 Department of Molecular and Cellular Neuroscience, Dorris Neuroscience Center, The Scripps Research Institute, La Jolla, CA-92037, USA; 7 Japan Agency for Medical Research and Development, Core Research for Evolutional Science and Technology (AMED-CREST), Chiyoda-ku, Tokyo 100-0004 Japan

## Abstract

The lipid mediator lysophosphatidic acid (LPA) signals via six distinct G protein-coupled receptors to mediate both unique and overlapping biological effects, including cell migration, proliferation and survival. LPA is produced extracellularly by autotaxin (ATX), a secreted lysophospholipase D, from lysophosphatidylcholine. ATX-LPA receptor signaling is essential for normal development and implicated in various (patho)physiological processes, but underlying mechanisms remain incompletely understood. Through gene targeting approaches in zebrafish and mice, we show here that loss of ATX-LPA_1_ signaling leads to disorganization of chondrocytes, causing severe defects in cartilage formation. Mechanistically, ATX-LPA_1_ signaling acts by promoting S-phase entry and cell proliferation of chondrocytes both *in vitro* and *in vivo*, at least in part through β1-integrin translocation leading to fibronectin assembly and further extracellular matrix deposition; this in turn promotes chondrocyte-matrix adhesion and cell proliferation. Thus, the ATX-LPA_1_ axis is a key regulator of cartilage formation.

Lysophosphatidic acid (LPA) is produced in the extracellular milieu such as in plasma, mainly by autotaxin (ATX), and acts on at least six receptors that are specific to LPA (LPA_1–6_) to exert its functions^1^,^2^. Historically, LPA was identified as a growth factor in serum. Indeed, LPA has been shown to stimulate the proliferation of many cell types including fibroblasts and cancer cells^3^,^4^. Especially, LPA was shown to promote cell cycle entry, i.e., promote the G_0_/G_1_- to S-phase transition^4^, although different cell types can respond in other ways such as with cycle exit for neural progenitor cells^5^. The molecular mechanisms underlying LPA-induced cell cycle progression as well as its *in vivo* relevance remain unclear. In constitutive LPA receptor knockout (KO) mice, only LPA_1_ KO mice showed obvious physical defects, including retardation of physical growth, prominent craniofacial abnormalities (including a shorter snout) and shorter limbs^6^,^7^. ATX is a secreted lysophospholipase D (lysoPLD) which catalyzes a reaction to produce LPA from lysophosphaditylcholine (LPC)^8^. ATX KO mice are embryonic lethal around E9.5–10.5 because of vascular defects^9^. ATX was originally identified as a cell motility-stimulating factor secreted by human melanoma cells^10^,^11^. Because enhanced expression of ATX has been demonstrated in various tumor tissues^12^, ATX may promote proliferation and migration of cancer cells through LPA production. ATX is also expressed in various normal tissues and is present at high concentration in various biological fluids. Thus, ATX has an important role in cell proliferation not only in cancer cells but also in various normal cell types. Here, we show that ATX-LPA-LPA_1_ signaling promotes the G_0_/G_1_-phase to S-phase transition in chondrocytes by enhancing integrin-dependent fibronectin assembly. These changes contribute to the normal development of cartilage tissues, based on analyses of both fish and mice.

## Results

### Loss of ATX-LPA_1_ signaling results in dyschondroplasia in zebrafish

**Figure 1:**
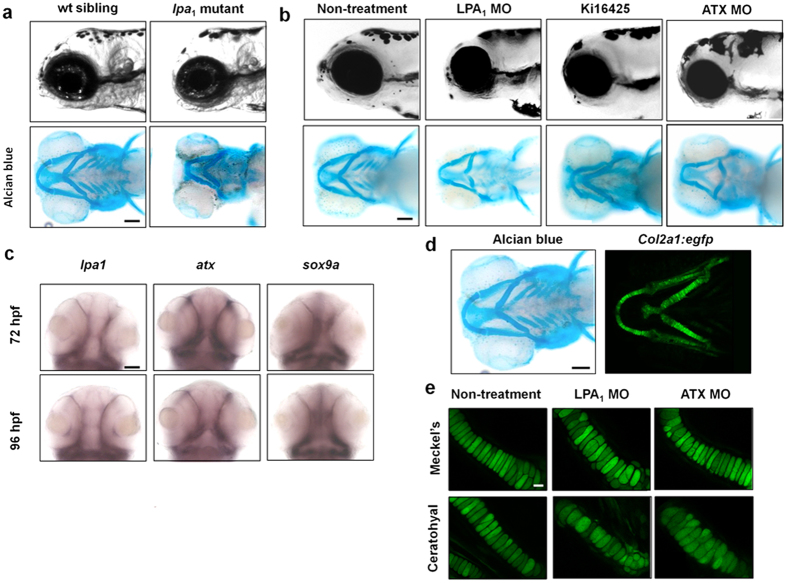
Loss of ATX-LPA_1_ signaling resulted in dyschondroplasia in zebrafish. (**a,b**) Loss of ATX- LPA_1_ signaling leads to deformation of the cephalic region in zebrafish embryos. Cephalic regions of wt and *lpa*_*1*_ mutant zebrafish embryos (**a**) and cephalic regions of zebrafish embryos treated with LPA_1_ antagonist, LPA_1_ or ATX morpholinos (**b**) at 96 hpf (side view). Cartilage tissues visualized by alcian blue and alizarin red staining are also shown (ventral view). Scale bar: 100 μm. (**c**) Both *lpa1* and *atx* are co-expressed with *sox9*, a marker of chondrocytes, in zebrafish embryo both at 72 and 96 hpf as judged by *in situ* hybridization. Scale bar: 50 μm. (**d,e**) Loss of ATX-LPA_1_ signaling in zebrafish embryos leads to mislocalization of chondrocytes in cartilage tissues. (**d**) EGFP is expressed specifically in chondrocytes in *col2*:*egfp* transgenic zebrafish at 120 hpf. Scale bar: 100 μm. (**e**) In zebrafish embryos treated with LPA_1_ or ATX morpholinos, chondrocytes are unevenly distributed in Meckel’s and ceratohyal cartilages at 120 hpf. Scale bar: 100 μm.

LPA-related genes are highly conserved in vertebrates. In zebrafish and mice, the amino acid sequences of ATX and LPA_1_ are 67 and 85% identical, respectively. The genes for ATX and the six LPA receptors in vertebrates are completely conserved in zebrafish^13^,^14^. We employed TILLING^15^ and identified a zebrafish *lpa*_1_ mutant with a vu374 mutation at G309 that results in a premature stop codon (Fig. S1a) in the first extracellular loop of LPA_1_. Homozygous *lpa*_1_ mutants are able to reach adulthood and are fertile but display a craniofacial malformation: a round-shaped cephalic region that is also phenocopied in *lpa*_1_ mutant mice^6^ (Fig. 1a).

A search for other mutant and morphant zebrafish with similar phenotypes identified several cases, e.g. the *foxe1* mutant. In most of these cases, cartilage formation was impaired^16^,^17^. In addition, *lpa*_*1*_ mRNA was highly expressed at 72 and 96 hour post fertilization (hpf) in the zebrafish embryos, and the expression pattern overlapped with that of *sox9a* mRNA (Fig. 1c), a marker of chondrocytes, which shows that chondrocytes express *lpa*_1_ in the cartilage. Staining the cartilage of the wild type and *lpa*_1_ mutant with alcian blue revealed that the mutant embryos had disorganized jaw cartilage at both 96 (Fig. 1a) and 120 hpf (data not shown). Both Meckel’s and ceratohyal cartilages (Fig. S1b), the two main cartilages of the lower jaw, were deformed. The lengths of Meckel’s and ceratohyal cartilages were shorter in the *lpa*_1_ mutants (Fig. 1a and Fig. S1c). Similar abnormalities were observed when LPA_1_ was down-regulated either by injection of a morpholino antisense oligonucleotide (MO) against LPA_1_^13^ or by treatment with an LPA_1_ antagonist, Ki16425 (Fig. 1b and Fig. S1d–f), which is known to be active against zebrafish LPA_1_^14^.

ATX is an LPA-producing enzyme that was found to be expressed in the cartilage (Fig. 1c). Knockdown of ATX with a high dose of ATX MO1 (2.5 ng) was previously shown to induce severe vascular defects^14^. When a low dose of ATX MO1 (0.3 ng) or ATX MO2 (3.2 ng) was injected, most of the embryos survived at 120 hpf but had rounded-shaped heads (Fig. 1b and Fig. S1e), similar to the heads of LPA_1_ mutant embryos. ATX morphant embryos also displayed impaired cartilage formation as illustrated by the loss of gill cartilage, *i.e.* deformation of Meckel’s and ceratohyal cartilages (Fig. 1b). We conclude that the loss of LPA_1_ signaling results in dyschondroplasia in zebrafish embryos and that ATX is the main LPA-producing enzyme in cartilage tissues.

To determine how loss of ATX-LPA_1_ signaling affects the behavior of chondrocytes in cartilage tissues, we employed *col2:EGFP* transgenic zebrafish, which expressed EGFP protein specifically in chondrocytes under the control of the *col2a1a* promoter^18^ (Fig. 1d). At 120 hpf, chondrocytes in both Meckel’s and ceratohyal cartilages maintained their intercalated and stacked organization in control embryos (Fig. 1d). In contrast, uneven sized and irregularly aligned chondrocytes were observed in LPA_1_ and ATX MO-injected embryos (Fig. 1e) as well as LPA_1_ antagonist (Ki16425)-treated embryos (data not shown).

The cartilage elements of the jaw are largely derived from cranial neural crest cells (CNCCs) that arise from dorsal and lateral regions of the neural ectoderm at 12 hpf and migrate into the area of the pharyngeal arches at 24–48 hpf, where the cells differentiate into chondrocytes to form the jaw cartilage at 72 hpf^17^,^19^. The expression patterns of *slug* and *sox10*, which are markers of CNCCs, were normal in both LPA_1_ and ATX morphant embryos (Fig. S1g), and the expression pattern of *sox9a* was also normal (data not shown). Thus, the migration of CNCCs and the maturation and differentiation of chondrocytes from CNCCs appeared to be unaffected by the loss of ATX-LPA_1_ signaling. It should be noted that bone tissues are not formed in zebrafish embryos until 120 hpf^19^, suggesting again that ATX-LPA_1_ signaling has a critical role in chondrogenesis.

### Loss of ATX-LPA_1_ signaling results in dyschondroplasia in mice

**Figure 2:**
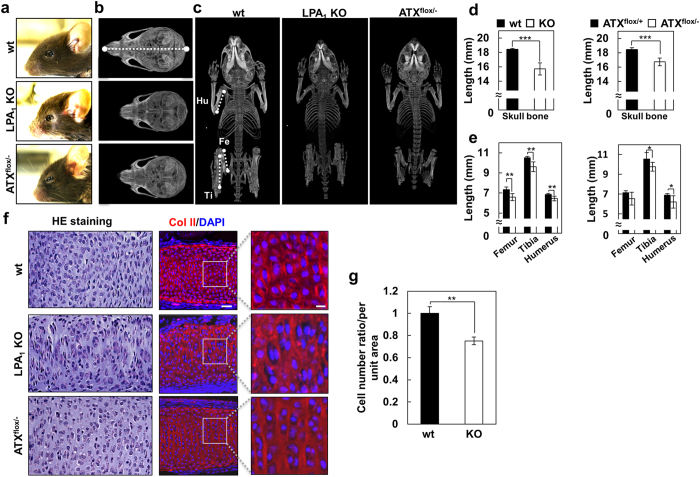
Loss of ATX-LPA_1_ signaling resulted in dyschondroplasia in mice. (**a–e**) Abnormal skeletal morphology in skull and limbs in LPA_1_ KO and ATX^flox/−^ mice. (**a**) The cephalic regions of wt, LPA_1_ KO and ATX^flox/−^ mice (side view) at 3 weeks of age. (**b,c**) Computed tomographic scanning images of skull and whole body bones. (**d,e**) Length of skull bone (**d**), femur, tibia and humerus (**e**) in wt, LPA_1_ KO, ATX^flox/+^ and ATX^flox/−^ mice at 3 weeks of age. (Data are mean ± s.d., n = 8–10, ^*^P < 0.05, ^**^P < 0.01, ^***^P < 0.001) (**f**) Loss of ATX-LPA_1_ signaling leads mislocalization of chondrocytes in mice. Sections of intersphenoid synchondrosis in wt, LPA_1_ KO and ATX^flox/−^ mice at P0 were stained with H&E or immunostained with anti-Col II antibody. Scale bar: 20 μm and 5 μm in magnified view. (**g**) The numbers of chondrocyte in intersphenoid synchondrosis per unit area of wt and LPA_1_ KO mice at P0, shown by relative ratio (Data are mean ± s.d., n = 4, ^**^P < 0.01).

We next examined the role of ATX-LPA_1_ signaling in cartilage formation in mice. LPA_1_ KO mice showed reduced anteroposterior growth of skull bone, and shorter femur, tibia and humerus (Fig. 2a–e). We focused on cartilage tissues in the cranial base (Fig. S2a) because the base is important for anteroposterior growth of skull bone. In fact, many mutant mice with defects in the base showed abnormal formation of skull bones like LPA_1_ KO mice^20^,^21^,^22^. We found that intersphenoid synchondrosis (the cartilage that links bones at the cranial base) ossified earlier in LPA_1_ KO mice at 3 weeks of age (Fig. S2b). At the cellular level, alignment of chondrocytes in the cartilage tissue was disturbed (Fig. 2f) and the number of cells was also significantly lower in LPA_1_ KO mice (Fig. 2g). Similar mislocalization of the chondrocytes was observed in other cartilage tissues such in the costa and femur (Fig. S2c).

Since global ATX KO mice are embryonic lethal because of impaired vascular formation^9^, we set out to produce conditional ATX KO mice. We produced mice with various combinations of ATX wild type, ATX-flox and null alleles, i.e., ATX^+/+^, ATX^+/flox^, ATX^flox/flox^, ATX^+/−^ and ATX^flox/−^ mice. We found that one flox allele insertion significantly decreased the serum ATX activity about 15% (Fig. S2d). Interestingly, mice with both ATX-flox and ATX-null alleles (ATX^flox/−^ mice) showed phenotypes similar to those of LPA_1_ KO mice. Because ATX^+/−^ mice as well as mice with other genotypes did not show obvious abnormality at all, it was speculated that the significant difference between ATX^+/−^ and ATX^flox/−^ (~50% minus ~35%, i.e., ~15%) was important for normal cartilage formation. Alternatively, it is possible that the insertion of flox allele impairs the transcription or stability of ATX pre-mRNA in a specific cell type, which affect the normal cartilage formation. From P0 to the adult stage, the ATX^flox/−^ mice displayed obvious abnormalities in craniofacial morphology and anteroposterior growth of the skull bone, and shorter femur, tibia and humerus (Fig. 2a–e). The intersphenoid synchondrosis ossified significantly earlier as well in the ATX^flox/−^ mice (Fig. S2b). In addition, at the cellular level, the alignment of chondrocytes was significantly disturbed in ATX^flox/−^ mice (Fig. 2f).

As was observed in zebrafish, loss of ATX-LPA_1_ signaling did not affect the differentiation of chondrocytes, since LPA_1_ KO did not affect the expressions at P0 of type II collagen (Col II), a marker of resting and proliferating chondrocytes or type X collagen (Col X), a marker of pre-hypertrophic and early hypertrophic chondrocytes (Fig. S2a,e). Taking account of the fact that both LPA_1_ and ATX were highly expressed in the cartilage tissues in both zebrafish and mice (Fig. 1c and Fig. S2f), we conclude that ATX-LPA_1_ signaling functions after chondrocyte differentiation and that dyschondroplasia is the main defect of LPA_1_ KO and ATX^flox/−^ mice, and is the cause of craniofacial abnormalities and retardation of physical growth in these mice.

### Inhibition of ATX-LPA_1_ signaling delays S-phase entry in chondrocytes

**Figure 3:**
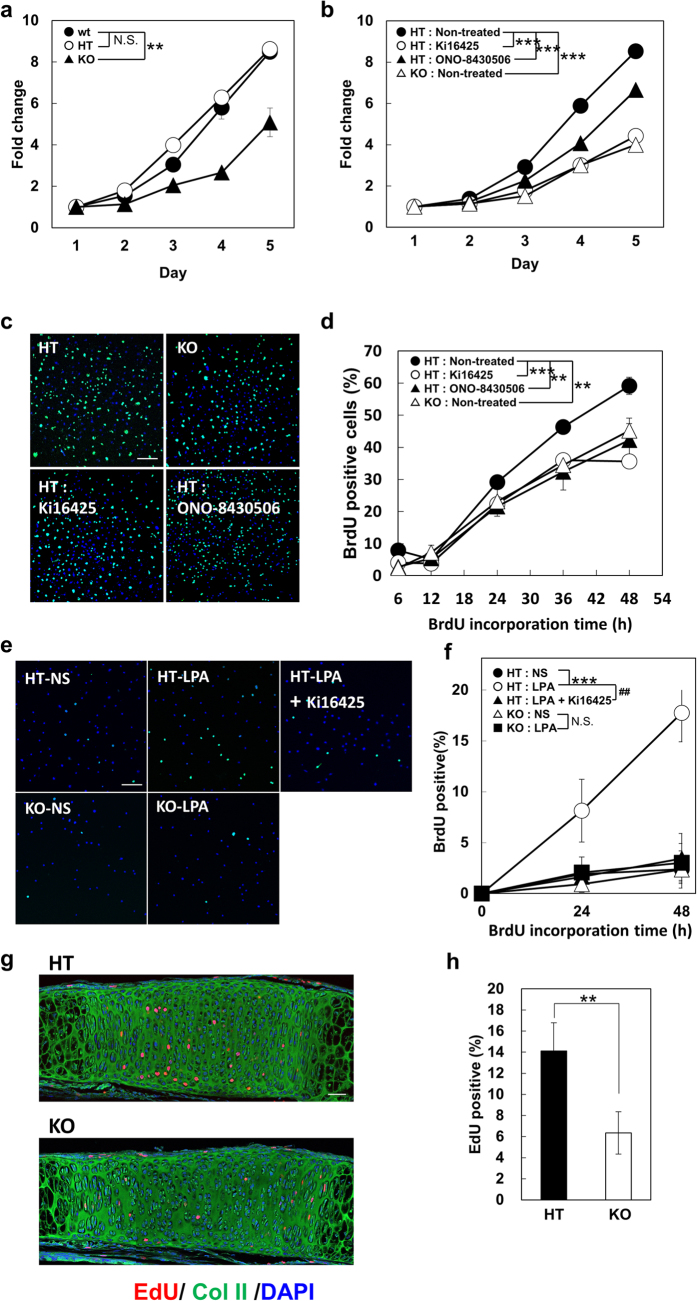
Inhibition of ATX-LPA_1_ signaling leads delayed S-phase entry in chondrocytes. (**a–f**) Role of LPA_1_ signaling in chondrocyte cell proliferation *in vitro*. (**a**) Chondrocytes isolated from LPA_1_^+/+^ (wt), LPA_1_^+/−^ (HT) and LPA_1_^−/−^ (KO) mice were cultured in medium containing 10% FCS and time-dependent cell proliferation was determined by cell counting kit-8 (Data are mean ± s.d., n = 3, N.S.: not significant, **P < 0.01 for day2–5). (**b**) HT chondrocytes were cultured as in (**a**) in the presence of LPA_1_ antagonist (Ki16425) or ATX inhibitor (ONO-8430506) (Data are mean ± s.d., n = 3, ^***^P < 0.001 for day 5, the significant differences were detected from day 3 in all comparisons). (**c–f**) LPA promotes S-phase entry of chondrocytes. After 24 hr starvation, chondrocytes were stimulated with 10% FCS (**c,d**) or 10 μM LPA (**e,f**) and time course dependent incorporation of BrdU was determined by immunofluorescence using anti-BrdU antibody. For HT chondrocytes, cells were also cultured in the presence of LPA_1_ antagonist (Ki16425) or ATX inhibitor (ONO-8430506) (NS: non-stimulated, Data are mean ± s.d., n = 3 (**c,d**) or 4 (**e,f**), N.S.: not significant, ^##^*P* < 0.01, ^**^P < 0.01, ^***^P < 0.001 for 48 hr, the significant differences were detected from 36 hr in all comparisons in **d**. Scale Bar: 100 μm. (**g,h**) Role of LPA_1_ signaling in chondrocyte cell proliferation *in vivo*. Cell proliferation of chondrocytes in intersphenoid synchondrosis of HT and KO mice were evaluated by immunofluorescent images of EdU incorporation (g) (Scale bar: 50 μm). The numbers of EdU positive chondrocytes in resting zone were also counted and data were shown as percentage toward total cell numbers (h). (Data are mean ± s.d., n = 4, ^**^P < 0.01).

To examine the effect of LPA signaling on cellular functions of chondrocytes, we established primary cultured chondrocytes from rib cages. We found that proliferation of LPA_1_^−/−^ chondrocytes was significantly slower than that of LPA_1_^+/+^ and LPA_1_^+/−^ chondrocytes (Fig. 3a). In addition, the cell size of LPA_1_^−/−^ chondrocytes was much smaller (Fig. S3a). We didn’t observe any significant changes in the proliferative activity (Fig. 3a) or cell size (data not shown) between LPA_1_^+/+^ and LPA_1_^+/−^ chondrocytes. Thus in the following experiments, we used LPA_1_^+/−^ chondrocytes as a control. Time-lapse images of the proliferating chondrocytes revealed that the doubling time for LPA_1_^−/−^ chondrocytes (1660 ± 400 min) was significantly longer than that for LPA_1_^+/−^ chondrocytes (1280 ± 220 min) (Fig. S3b and Videos 1 and 2). Although the duration of the M phase did not differ between the two types of chondrocytes (Fig. S3c and Videos 1 and 2), we found that cell cycle progression from the G_0_/G_1_ to S-phase as judged by BrdU incorporation was significantly reduced in LPA_1_^−/−^ chondrocytes (Fig. 3c,d). Importantly, the smaller cell size and the reduced proliferative activity were reproduced by adding an LPA_1_ antagonist (Ki16425) to cultures of LPA_1_^+/−^ chondrocytes (Fig. 3b–d, Fig. S3a,b and Video 3) or LPA^+/+^ chondrocytes (data not shown), indicating that the phenotypes of LPA_1_^−/−^ chondrocytes are not due to congenital changes of the chondrocytes. Addition of an ATX inhibitor (ONO-8430506) also resulted in a smaller cell size (Fig. S3a) and reduced proliferative activity, albeit to a lesser extent than the LPA_1_ antagonist (Fig. 3b–d and Video 4). Unlike LPA_1_^−/−^ chondrocytes or chondrocytes treated with LPA_1_ antagonist or ATX inhibitor, chondrocytes from ATX^flox/−^ mice proliferated normally. It should be mentioned that the culture medium contained significant amounts of ATX and its substrate, lysophosphatidylcholine, both of which are present within fetal calf serum (FCS).

In serum-free medium, LPA significantly stimulated S phase entry of LPA_1_^+/−^ chondrocytes whereas the effect was not observed in LPA_1_^−/−^ chondrocytes or Ki16425-treated LPA_1_^+/−^ chondrocytes (Fig. 3e,f). *In vivo* imaging of LPA_1_ KO mice at P0 using 5-ethynyl-2′-deoxyuridine (EdU) showed that the proliferation of chondrocytes in the intersphenoid synchondrosis was significantly decreased (Fig. 3g,h). These *in vivo* and *in vitro* findings indicate that in the absence of LPA_1_ signaling, the G_0_/G_1_-to-S phase transition is prolonged, which explains the reduction of cell proliferation and decreased cell number of chondrocytes in the intersphenoid synchondrosis (Fig. 2g and 3g, h).

### Integrin-mediated adhesion to fibronectin promotes LPA-induced S-phase entry of chondrocytes

**Figure 4:**
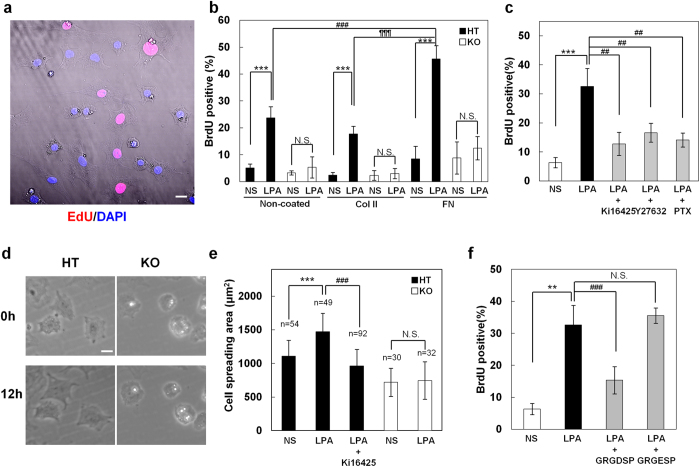
LPA-induced S-phase entry is enhanced by fibronectin and is integrin-dependent in chondrocytes. (**a**) Chondrocytes that enter the S-phase as judged by EdU incorporation spread fully. Merged phase and confocal image of LPA-stimulated HT chondrocytes on cell culture-treated (non-coated) plates 48hr after stimulation. Nuclei colored in red indicate EdU positive cells. Scale bar: 20 μm. (**b,c**) LPA-induced S-phase entry is enhanced by the presence of fibronectin, is LPA_1_-dependent and is mediated via both G_α12/13_ and G_αi_ pathways. (**b**) LPA-induced S-phase entry of HT and KO chondrocytes seeded on non-, Col II- and FN-coated plates was evaluated by BrdU incorporation assay (NS: non-stimulated, Data are mean ± s.d., n = 4, N.S.: not significant, ^***^P < 0.001, ^###^*P* < 0.001, ^¶¶¶^*P* < 0.001). (**c**) Effects of LPA_1_ signal inhibitors (LPA_1_ antagonist (Ki16425), ROCK inhibitor (Y27632) or PTX (G_αi_ inhibitor)) on LPA-induced S-phase entry in the presence of fibronectin (NS: non-stimulated, Data are mean ± s.d., n = 4, ^***^P < 0.001, ^##^*P* < 0.01). (**d,e**) LPA stimulates cell spreading via LPA_1_. (**d**) Time lapse images of LPA-stimulated HT and KO chondrocytes at 0 and 12 hr after LPA stimulation on FN-coated plates. Scale bar: 20 μm. (**e**) HT and KO chondrocytes were stimulated with LPA and evaluated the cell spreading area 12 hr after the stimulation (NS: non-stimulated, Data are mean ± s.d., N.S.: not significant, ^***^P < 0.001, ^###^*P* < 0.001). (f) LPA-induced S-phase entry is integrin-dependent. LPA-induced S-phase entry of HT chondrocytes was evaluated by BrdU incorporation in the presence of integrin blocking peptide (GRGDSP) and control peptide (GRGESP) (NS: non-stimulated, Data are mean ± s.d., n = 4, N.S.: not significant, ^***^P < 0.001, ^###^*P* < 0.001).

When stimulated by LPA in the presence of EdU, most of the EdU-positive chondrocytes adhered tightly to the culture plates and spread fully (Fig. 4a), suggesting that the adhesive property of the chondrocytes affected their proliferation. Since chondrocytes in cartilage tissues are surrounded by and are in contact with extracellular matrix (ECM), which mainly consists of Col II and fibronectin (FN), we examined the effect of ECM on LPA-induced cell proliferation. Strikingly, FN coating dramatically enhanced the LPA-induced BrdU incorporation of LPA_1_^+/−^ chondrocytes (Fig. 4b). The FN effect was not observed in LPA_1_^−/−^ chondrocytes and was suppressed by treating the cells with LPA_1_ antagonist, Ki16425, as well as by treating them with Y27632 and PTX (Fig. 4b,c), indicating that LPA-induced proliferation on FN-coated plates was LPA_1_-dependent and mediated via both the G_α12/13_ and G_αi_–linked pathways. LPA enhanced the spreading of LPA_1_^+/−^ but not LPA_1_^−/−^ chondrocytes on FN-coated plates (Fig. 4d,e). LPA had similar effects on Col II-coated plates and on non-coated plates (data not shown) (Fig. 4b and Fig. S4a,b). On both FN- and Col II-coated plates, cell proliferation was suppressed by GRGDSP, an integrin blocking peptide, but not by a control peptide, GRGESP (Fig. 4f and Fig. S4c), showing an important role for integrins in LPA-induced chondrocyte proliferation.

### LPA enhances fibronectin assembly through LPA_1_

**Figure 5:**
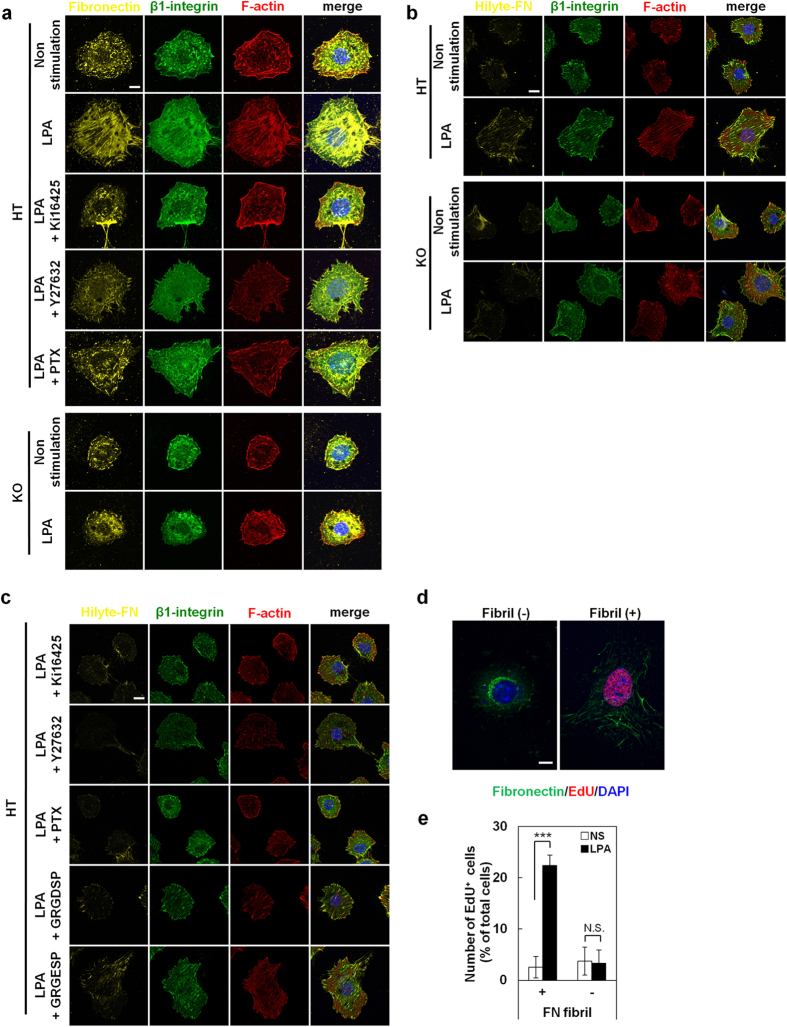
LPA enhances formation of fibronectin fibrils through LPA_1_ leading to S-phase entry of chondrocytes. (**a**) LPA enhances formation of fibronectin fibrils through LPA_1_. HT chondrocytes were stimulated with LPA in the presence of LPA_1_ antagonist (Ki16425), ROCK inhibitor (Y27632) or PTX (G_αi_ inhibitor). Cells were also immunostained with anti-β1-integrin antibody and phalloidin. Scale bar: 5 μm. (**b,c**) LPA enhances the assembly of extracellularly-added FN into FN fibrils via LPA_1_, G_α12/13_, G_αi_ and integrin-mediated signaling. (**b**) Intracellular distribution of fluorescent-labeled FN (Hilyte488-FN) added in LPA-stimulated chondrocytes. Cells were also immunostained with anti-β1-integrin antibody and phalloidin. Scale bar: 5 μm. (**c**) Chondrocytes were stimulated with LPA in the presence of Hilyte-488 FN and LPA_1_ antagonist (Ki16425), ROCK inhibitor (Y27632), PTX (G_αi_ inhibitor), integrin blocking peptide (GRGDSP) or control peptide (GRGESP). Scale bar: 5 μm. (**d,e**) FN distributes in filamentous structures (FN fibrils) in chondrocytes that undergo LPA-stimulated S-phase entry. (**d**) HT chondrocytes were stimulated with LPA in the presence of EdU on Col-II coated plates and were immunostained with anti-FN antibody. Scale bar: 5 μm. (**e**) Numbers of EdU-positive cells with or without FN fibrils both in non-stimulated and LPA-stimulated chondrocytes. (Data are mean ± s.d., n = 3, N.S.: not significant, ^***^P < 0.001).

FN plays an important role in cell adhesion, which, in turn, affects the proliferation and survival of many cell types^23^. In LPA_1_^+/−^ chondrocytes at 24 hours after LPA stimulation, FN was distributed in filamentous structures, which overlapped with F-actin and β1-integrin (Fig. 5a). In contrast, in LPA_1_^−/−^ chondrocytes and LPA_1_^+/−^ chondrocytes treated with Ki16425, Y27632 or PTX, such filamentous structures were less developed (Fig. 5a). In the presence of LPA, extracellularly added fluorescently labeled FN (Hilyte-488 FN) was readily incorporated into the filamentous structures and colocalized with F-actin and β1-integrin in LPA_1_^+/−^ but not LPA_1_^−/−^ chondrocytes (Fig. 5b). It thus appears that FN, once secreted from chondrocytes, is incorporated and assembled into filamentous structures in an LPA_1_- and integrin-dependent manner. Consistent with this notion, LPA-induced FN assembly was prominently suppressed by Ki16425, Y27632 and PTX, and partially inhibited by GRGDSP peptide but not by GRGESP (Fig. 5c). Importantly, most of the EdU-positive cells showed the filamentous FN structures (Fig. 5d,e). On the basis of these observations, we hypothesized that LPA stimulates S-phase entry by enhancing FN assembly downstream of LPA_1,_ through β1-integrin activation. To further confirm that the interaction of β1 integrin and FN transmits the intracellular signaling, we examined the formation of focal adhesions which are known to be activated by the coordinated action of β1-integrin and FN. We observed that LPA_1_ signaling promoted focal adhesion assembly as judged by colocalization of vinculin and actin (Fig. S5). Focal adhesions distributed peripherally in non-stimulated cells even in LPA_1_^−/−^ chondrocytes. But, only in LPA_1_^+/−^ chondrocytes, LPA promoted focal adhesions larger and some of them existed under the cell bodies. and this conformational change was also inhibited by Ki16425, Y27632 and PTX (Fig. S5). These observations confirmed the idea that intracellular signaling via β1-integrin was activated downstream of LPA_1_ signaling.

### ECM formed through LPA_1_ signaling supports the proliferation of chondrocytes

**Figure 6:**
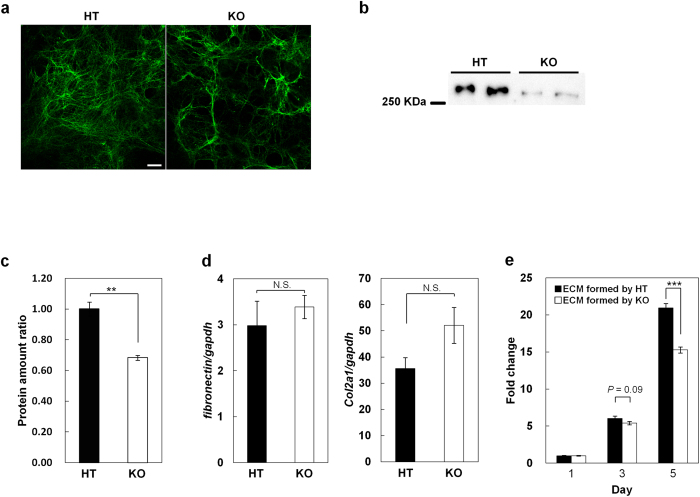
ECM formed through LPA_1_ signaling supports the proliferation of chondrocytes. (**a,b**) FN deposition is enhanced by LPA_1_ signaling. (**a**) Chondrocytes were cultured in medium containing 10% FCS for 10 days and immunostained with anti-FN antibody. Scale bar: 10 μm. (**b**) Deoxycholate-insoluble fibronectin in the extracellular matrix (ECM) was detected by western blot. (**c**) Comparison of ECM amount between HT and KO chondrocytes. (**d**) FN and Col II are similarly expressed in HT and KO chondrocytes. (Data are mean ± s.d., n = 3, N.S.: not significant) (**e**) ECM formed through LPA_1_ signaling supports the cell proliferation efficiently. HT chondrocytes were cultured on the decellularized-ECM plates which formed either by HT chondrocytes (ECM formed by HT) or KO chondrocytes (ECM formed by KO), and time-dependent cell proliferations were determined. (Data are mean ± s.d., n = 3, ^***^P < 0.001).

To examine the biological significance of LPA_1_-mediated FN assembly, we prepared ECM by culturing LPA_1_^+/−^ and LPA_1_^−/−^ chondrocytes. After 10 days of culture, filamentous FN structures were well developed in LPA_1_^+/−^ chondrocytes but less developed in LPA_1_^−/−^ chondrocytes (Fig. 6a). Furthermore, LPA_1_^−/−^ chondrocytes had significantly less deoxycholate-insoluble FN protein (Fig. 6b) and significantly less total ECM proteins (Fig. 6c). Under these conditions, both types of chondrocytes showed equal cell numbers per well (Fig. S6a) and FN and Col II mRNA levels (Fig. 6d), suggesting that deposition of ECM components was attenuated in LPA_1_^−/−^ chondrocytes. Transmission electron micrographs of LPA_1_^+/−^ and LPA_1_^−/−^ chondrocytes confirmed that thick filamentous bundles were better developed in LPA_1_^+/−^ chondrocytes than in LPA_1_^−/−^ chondrocytes (Fig. S6b). We next compared the abilities of decellularized ECMs (Fig. S6c) formed by LPA_1_^+/−^ and LPA_1_^−/−^ chondrocytes to support cell proliferation of LPA_1_^+/−^ chondrocytes. After 5 days of culture, ECMs formed by LPA_1_^+/−^ chondrocytes had significantly more cells than ECMs formed by LPA_1_^−/−^ chondrocytes (Fig. 6e). Thus LPA_1_ signaling regulates the nature of the ECM and provides the proper external milieu for cell proliferation.

## Discussion

LPA_1_ KO mice show obvious craniofacial abnormalities, retardation of physical growth and a low bone mass^6^,^7^. Previous reports indicated that loss of LPA_1_ signaling resulted in impaired differentiation of osteoclasts and osteoblasts *in vitro*^7^,^24^,^25^. However, the fundamental causes of the defects of LPA_1_ KO mice has remained unclear. In this study we showed that LPA_1_ is highly expressed in chondrocytes in both fish (Fig. 1c) and mice (Fig. S2f), and has a critical role in stimulating the proliferation and positioning of chondrocytes (Fig. 1e and 2f), thereby promoting proper cartilage formation. Bones, especially long limb bones, are principally formed by a process called endochondral ossification, in which cartilage is replaced by bone and chondrocytes serve as a center of bone growth^26^. Because the cartilage phenotype was observed before bone tissues had formed, we conclude dyschondroplasia is the primary cause of impaired bone development and retarded physical growth of LPA_1_ KO mice. We also showed that ATX is highly expressed in chondrocytes of both zebrafish and mice, and that inactivation of ATX in both species phenocopied dyschondroplasia as observed when LPA_1_ was attenuated or deleted. We therefore conclude that ATX is the major LPA-producing enzyme in cartilage tissues.

The dyschondroplasia of LPA_1_ KO and ATX^flox/−^ mice can be attributed to dysfunction of chondrocytes. *In vitro* experiments revealed that the ATX-LPA_1_ axis has a pivotal role in chondrocyte proliferation. We also examined the effect of an ATX inhibitor and LPA_1_ antagonist on cell proliferation of various cell types and found that chondrocytes are more sensitive to LPA_1_ signaling than other cell types (data not shown). The ATX-LPA_1_ axis promoted cell cycle progression of chondrocytes, in agreement with LPA having growth factor-like activities^3^,^4^,^27^,^28^ and anti-apoptoc effects as observed in the nervous system^5^,^29^,^30^. Pathophysiological effects of LPA as a growth factor remain less clear, however it can disrupt normal processes relevant to neurodevelopmental disorders like hydrocephalus and schizophrenia^31^,^32^. To the best of our knowledge, the present study is the first to demonstrate a physiological meaning for LPA-induced cell proliferation through cell cycle progression.

Our present results raise a new question: how does LPA support the proliferation of chondrocytes downstream of LPA_1_? Kingsbury *et al*. reported that LPA-induced cell growth of neural progenitor cells is not due to increased proliferation but rather to reduced cell death via LPA_1/2_^5^. However, this is not the case, because we did not observe any sign of cell death when LPA_1_ signaling was attenuated (Videos 1,2,3,4). These observations also exclude the possibility that loss of LPA_1_ signal induced anoikis in chondrocytes. Clues to answering this question are that LPA_1_^−/−^ chondrocytes were less adhesive to ECM than LPA_1_^+/−^ chondrocytes (Fig. 4d,e), and that LPA-induced chondrocyte proliferation was dramatically enhanced by the presence of FN and suppressed by an integrin-blocking RGD peptide (Fig. 4b,f). These findings suggest that LPA supports chondrocyte proliferation by upregulating β1-integrin-mediated cell adhesion to FN. Indeed, mice in which cartilage-specific β1-integrin is conditionally knocked out exhibited dyschondroplasia similar to that of LPA_1_ KO mice^33^. Furthermore, topical administration of anti-α5β1-integrin antibody or RGD-containing peptide to the upper limbs of mouse fetuses suppressed chondrocyte proliferation and shortened the upper limbs^34^. Together, these findings indicate that β1-integrin and LPA_1_ have closely related roles in the formation of cartilage.

**Figure 7:**
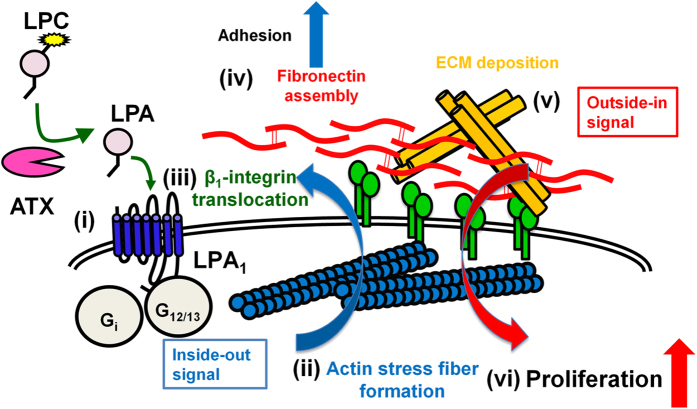
Diagram of ATX-LPA-LPA_1_ axis-induced cell proliferation via integrin-dependent fibronectin assembly in an inside-outside-in manner. In cartilage tissues, LPA produced by ATX activates LPA_1_ in chondrocytes (i); LPA_1_ signaling induces actin stress fiber formation possibly through G_α12/13_ and G_αi_ (ii); β1-integrin is translocated along with actin stress fibers (iii); the translocated β1-integrin then induces fibronectin assembly (fibronectin fibrils) (iv); fibronectin fibrils promote ECM deposition (v); ECM thus forms and then supports the proliferation of chondrocytes by promoting cell adhesion (vi). All these processes contribute to normal cartilage formation.

Another important observation for understanding the mechanism of LPA-induced proliferation is that FN was assembled to form filamentous structures (Fig. 5a–c and Fig. S5). We presumed that the filamentous FN structure is a multimeric FN, i.e., FN fibril. FN fibrils within the ECM play central roles in both physiological and pathological processes during development and tissue regeneration by coordinating cell adhesion, growth, migration and differentiation. FN is assembled to form FN fibrils in an integrin- and actin stress fiber-dependent manner^35^,^36^,^37^,^38^. *In vitro* studies using fibroblasts and platelets have suggested that integrins and some agonists for G protein–coupled receptors including LPA are involved in the assembly of FN fibrils^39^,^40^,^41^. The present results clearly demonstrate that LPA_1_ signaling induced assembly of the filamentous FN structures undersurface of chondrocytes; the FN structures then modified the proliferative and adhesive property of the cells. The structures were co-localized with actin stress fiber and β1-integrin (Fig. 5a,b) and their formation was canceled by Y27632 and PTX (Fig. 5a,c). The coordinated changes in integrins and the actin cytoskeleton observed here parallel cadherin and focal adhesion assembly associated with actin changes observed in Schwann cells of the nervous system^42^. All together, these findings support the idea that the filamentous FN structure is an FN fibril and that the LPA-LPA_1_ axis regulates actin stress fiber formation and then β1-integrin translocation. These changes stimulate the FN assembly to form FN fibrils, which enhances the proliferation of chondrocytes (Fig. 7). This model is further supported by the observation that the ECM formed by chondrocytes in the presence of LPA_1_ signaling supports cell proliferation more efficiently than ECM formed in the absence of LPA_1_ signaling (Fig. 6b). The observation also implicates that integrin activity itself can be regulated in both an inside-out and outside-in manner^43^,^44^. The present results indicate that the ATX-LPA_1_ axis regulates integrins in an inside-out manner (from step (i) to (v) in Fig. 7), which results in the formation of FN fibrils and the subsequent organization of other ECM components such as Col II. The ECM thus formed then regulates integrins in an outside-in manner (from step (v) to (vi) in Fig. 7), which contributes to proper cell adhesion and proliferation of chondrocytes, leading to normal cartilage development. Thus, in addition to the previously indicated LPA_1_-G_αi_-Akt pathway^45^, we propose a new model of ATX-LPA-LPA_1_ axis-induced cell proliferation that is mediated by integrin-dependent fibronectin assembly, occurring in an “inside-outside-in” manner (Fig. 7).

A key question is whether cells other than chondrocytes utilize this system for their proliferative activity. Chondrocytes and fibroblasts are both derived from mesenchymal stem cells. LPA_1_ KO mice were resistant to bleomycin-induced lung fibrosis^46^ and unilateral ureteral obstruction-induced renal fibrosis^47^. Thus it is possible that LPA-LPA_1_ signaling upregulates proliferation of fibroblasts in a manner similar to what we propose in chondrocytes. In addition, because LPA_1_ and ATX are overexpressed in several cancers^8^,^48^,^49^, the enhanced proliferation of certain LPA_1_-positive cancer cells, such as glioblastoma cells^50^, may reflect a similar mechanism, which is currently being examined.

## Materials and Methods

### Reagents

Lysophospholipids including 1-oleoyl (18:1)-LPA and 1-myristoyl (14:0)-LPC were purchased from Avanti Polar Lipids. Lysophospholipids were dried under nitrogen gas and dissolved in 0.1% fatty acid-free bovine serum albumin (Sigma-Aldrich)-PBS using water bath sonication and stocked in −20 °C. Y27632, ROCK inhibitor, was purchased from Merck. Pertussis toxin was purchased from Wako Pure Chemicals, GRGDSP and GRGESP were purchased from AnaSpec. Ki16425 was chemically synthesized as described previously^51^. ATX inhibitor, ONO-8430506 was a generous gift from Ono pharmaceutical Co., Ltd^52^.

### Zebrafish lines

Zebrafish were maintained according to the Guidelines for Animal Experimentation of Tohoku University and the protocol was approved by the Institutional Animal Care and Use Committee at Tohoku University. Wild-type AB zebrafish were obtained from Zebrafish International Resource Center (University of Oregon, Eugene, OR). The LPA_1_ mutant was generated by target-selected mutational inactivation of genes (TILLING) according to standard methods^53^. Fish were maintained at 27–28 °C under a controlled 13.5 h light/10.5 h dark cycle. Embryos were obtained from natural spawning and staged according to morphology. The standard staging of zebrafish embryos is used and determined in hpf (hour post fertilization) or dpf (day post fertilization) at 28 °C^54^.

### Generation of *col2a1:egfp* transgenic lines

To generate a *col2a1*:*egfp* transgenic line, the *col2a1* promoter^18^ was introduced upstream of *egfp* using the Tol2kit system^55^. Briefly, the *col2a1* promoter was introduced into p5E-MCS vector, and then p5E-*col2a1*, pME-EGFP, p3E-polyA and pDestTol2pA2 were combined with LR clonase II plus (Life Technologies). Twenty-five ng of the DNA plasmid was injected into the embryos at the 1-cell stage to establish a *col2a1*:*egfp* transgenic line that selectively expresses EGFP in cartilage.

### Microscopic Analysis

Zebrafish larvae were anesthetized with 0.016% tricaine methanesulfonate solution (Sigma-Aldrich) and positioned in 3% methylcellulose (Sigma-Aldrich) on a slide glass. Images were captured with a Leica M80 stereomicroscope equipped with a Leica DFC425 digital camera (Leica Microsystems). *Col2a1:egfp* transgenic fish larvae were positioned in 1.5% agarose (Sigma-Aldrich) on a glass-bottom well (MatTek, MA) and imaged with a LSM 700 confocal laser-scanning microscope (Carl Zeiss).

### Alcian blue staining

Zebrafish cartilage and bone were double stained with alcian blue and alizarin red as described previously^56^. At 4 or 5 dpf, larvae were fixed with 4% PFA in PBS, rocked at room temperature for 2 h, washed and dehydrated with 50% EtOH at room temperature for 10 min. After removing the EtOH, the larvae were double stained with acid-free stain solution (0.02% alcian blue, 0.005% alizarin red, 150 mM MgCl_2_ in 70% EtOH), rocked overnight at room temperature, washed with water, depigmented with bleaching solution (1.5% H_2_O_2_ in 1% KOH), rocked at room temperature for 20 min, and cleared with successive changes of a solution of glycerol and KOH. The lengths of Meckel’s and ceratohyal cartilages were measured with a Zeiss Axio Imager (Carl Zeiss MicroImaging). Larvae with smaller or abnormally bent cartilage compared with control larvae were classified as ‘malformed’ larvae.

### Antisense morpholino injection

The morpholino (MO) sequences were:

LPA_1_ MO, 5′-TGGAGCACTTACCCAATACAATCAC-3′^13^;

ATX MO1 (MO1-enpp2), 5′-GGAGAATACCTGGGTCGAGACACCG-3′^14^;

ATX MO2 (MO2-enpp2), 5′-TTCTTTCAAAGTCCCTACCTTTGCA-3′^14^.

LPA1 (2.5 ng), ATX MO1 (0.3 ng) and ATX MO2 (3.2 ng) of each MOs were injected into the yolks of one- to two-cell stage embryos.

### Ki16425 treatment for zebrafish embryos

Embryos were treated with 120 μM of Ki16425 in the embryo medium with 1% DMSO^57^ from 48 hpf. Embryo medium containing Ki16425 was replaced approximately every 24 hours.

### Genotyping of *lpa*_*1*_ mutant

The *lpa*_*1*_ mutation was genotyped by restriction fragment length polymorphism (RFLP) analysis. All the primers were designed by a web-based program, dCAPS Finder 2.0^58^. The primer sequences were:

LPA_1_-180, 5′-ACAAGAAGGCTAACGGTTAGCACGTG-3′;

LPA_1_-181, 5′-ACAAGAAGGCTAACGGTTAGCAGGTG-3′;

LPA_1_-182, 5′-CATGACGATAGACATGGTCCAGATGATG-3′.

In the 198-bp PCR products derived from the wild-type allele with LPA_1_-180 and LPA_1_-182, an *Scr*FI restriction site was introduced, and therefore the *Scr*FI treatment degraded the 198-bp PCR product from the wild-type allele into a 174-bp fragment. The PCR products were cleaved with *Scr*FI and resolved on a 4% agarose gel. Similarly, the 198-bp PCR products from the mutant alleles with LPA_1_-181 and LPA_1_-182, *Hph*I restriction site were introduced. The *Hph*I restriction enzyme degrades the PCR product from mutant allele into a 163-bp DNA fragment.

### Whole mount *in situ* hybridization

Antisense RNA probes labeled with digoxigenin (DIG) for *lpa1, atx, slug, sox10* and *sox9a* were prepared with an RNA labeling kit (Roche Applied Science). Whole-mount *in situ* hybridization was performed as previously described^59^.

### Mice

Mice were maintained according to the Guidelines for Animal Experimentation of Tohoku University and the protocol was approved by the Institutional Animal Care and Use Committee at Tohoku University. The LPA_1_ KO mice generated by Contos *et al*.^6^ were transferred and maintained on a mixed 129SvJ/C57BL/6J background. Experiments comparing LPA_1_ KO and HT mice used offspring of mice heterozygous for the LPA_1_ mutant allele. The ATX conditional KO mice with two floxP alleles (ATX^flox/flox^) were generated by van Meeteren *et al*.^60^. ATX heterozygous KO mice (ATX^+/−^) were generated by Tanaka *et al*.^9^. Mice with a floxP allele and ATX null allele (ATX^flox/−^) were generated by crossing ATX^+/−^ and ATX^flox/flox^ mice.

### Skeletal analysis

Plain radiographs were taken using a soft X-ray apparatus (LaTheta LCT-200, Hitachi-Aloka). The size of the head and the lengths of the femur, tibia and humerus were determined with ORS Visual (Nihon Binary) software. Whole-mount skeletal staining was conducted as described previously, with slight modifications^61^. Briefly, mice were skinned, eviscerated and dehydrated in 95% (v/v) ethanol overnight. Skeletons were stained with 0.3% (w/v) alcian blue (Sigma-Aldrich) for 24 hours, stained with 1.5% (w/v) alizarin red (Wako) for 2 hours, treated with 1% (w/v) potassium hydrate and stored in glycerol/EtOH.

### Isolation of primary mouse rib chondrocytes

Chondrocytes were isolated as previously described^62^. In short, chondrocytes from rib were isolated from newborn wt, LPA_1_ HT or KO mice. Rib cages were dissected, rinsed in PBS, incubated at 37 °C for 45 min in 3 mg/ml collagenase type II (Worthington), cleaned of adherent tissues and digested with 0.5 mg/ml collagenase type II at 37 °C overnight. The cell suspension was filtered through a 40 μm cell strainer. The cells were washed, counted and plated. For all experiments, chondrocytes were plated 48 hr before any treatment.

### Evaluation of cell proliferation

Cell proliferation was determined with a Cell Counting Kit-8 (Dojindo). Cells were plated in 96-well plates at 1 × 10^4^ cells per well and cultured in the growth medium. Sixty min before the indicated time points, CCK-8 solution was added to each well and the cell numbers were measured by reading the absorbance (450 nm). To evaluate DNA synthesis, cells were seeded on cell culture-treated or Col II- or FN-coated (Sigma Aldrich, 5 μg/mL each) wells in 96-well plates. After 24 hr starvation, the cells were treated with 10 μM 5′-BrdU (Sigma-Aldrich) and 10% FCS or 10 μM LPA for the indicated times. When an inhibitor was used, cells were treated with Ki16425 (5 μM), ONO-8430506 (10 μM), Y27632 (10 μM), GRGDSP (500 μM) or GRGESP (500 μM) for 30 min before the addition of FCS or LPA. PTX (200 ng/mL) was treated at the same time as starvation. 5′-BrdU incorporation was quantified by counting 5′-BrdU-positive cells. 5′-BrdU was detected by anti-BrdU antibody conjugated to fluorescein (Roche, 1:1000). For EdU (Life Technologies) incorporation *in vitro*, 10 μM EdU was used instead of 5′-BrdU. To examine the incorporation of EdU *in vivo*, pups (P0-1) were subcutaneously injected with 50 mg/kg EdU and were sacrificed 2 hours after the injection. Heads of the embryos were dissected and fixed in 4% paraformaldehyde overnight at 4 °C. Tissues were embedded in Optimal Cutting Temperature (OCT) compound and stained according to the manufacturer’s instructions. EdU-positive cells were counted in a resting zone within a unit area. All images were captured with the LSM700 confocal microscope equipped with 10×/0.45 M27 Plan-Apochromat.

### Tissue and cell staining

For histological analysis, newborn mice were fixed in 4% fresh paraformaldehyde in PBS, pH 7.2, overnight, dehydrated in a graded alcohol series (50, 70, 90, 95, and 99.5%), and embedded in paraffin. Sections were cut at 10 μm and stained with hematoxylin and eosin (Mutoh Industries).

For immunohistochemistry, paraformaldehyde-fixed tissues were embedded in OCT compound or in paraffin. For paraffin sections, antigens were retrieved with 10 mM citrate buffer (pH 7.0) at 120 °C for 10 min. Tissue sections were cut with a cryostat (Leica Microsystems) or microtome (Leica Microsystems). Primary antibodies were used with predetermined optimal concentrations. The primary antibody was anti-Col II (Abcam, ab21291, 1:250). The secondary antibody to detect Col II was conjugated with Alexa Fluor 488 or 568 (Life Technologies, 1:200). Nuclei were counterstained with 4′, 6-diamidino-2-phenylindole (DAPI).

For Immunofluorescence, cells were plated on glass coverslips (Thermo Fisher Scientific) that had been treated with cell culture or coated with Col II or FN, and were fixed with 4% paraformaldehyde in PBS for 30 min, permeabilized with 0.5% TritonX-100 in PBS for 15 min, and blocked with 3% BSA/PBS for 30 min. The primary antibodies were goat anti-FN (Santa Cruz Biotechnology, sc-6952, 1:50), rabbit anti-vinculin (Abcam, ab73412, 1:150) and rat anti-β1-integrin (Millipore, MAB1997, 1:1000). The secondary antibodies for detecting goat anti-FN, rabbit anti-vinculin and rat anti-β1-integrin were conjugated with Alexa Fluor 488 or 647 (Life Technologies. 1:1000). Actin filaments were stained with Alexa594-conjugated phalloidin (Life Technologies, A12381, 1:250). Fluorescent images were captured with the LSM700 confocal microscope equipped with 63×/1.40 Oil DIC M27 Plan-Apochromat.

### Time-lapse

In live-cell imaging of chondrocytes, phase-contrast images were taken (LD plan-NEOFLUAR, 20x/0.4, Ph2, 6frames/hr) with a Zeiss inverted microscope (Axio Observer.Z1) equipped with a heated chamber (37 °C) and CO_2_ controller (5.0%) over a period of 48 hr. Primary chondrocytes were seeded onto 12-well plates (Greiner), incubated for 2 days at 37 °C and treated with an LPA_1_ antagonist (Ki16425, 5 μM) or an ATX inhibitor (ONO-8430506, 10 μM). Live-cell images were taken every 5 or 10 min for 48 hr. We chased cells that divided twice. The doubling time was taken as the time between the two divisions. The duration of the M phase was evaluated by cell morphology. Supplementary Videos 1,2,3,4 were simplified as 1.5 frames/hr for downsizing.

### Evaluation of cell spreading

Cells were plated on glass coverslips coated with Col II or FN and were stained with phalloidin. Cell images were captured with the LSM700 confocal microscope equipped with 63×/1.40 Oil DIC M27 Plan-Apochromat. and the cell spreading areas were calculated using the Zeiss Efficient Navigation system (Carl Zeiss).

### Quantitative RT-PCR

To prepare total RNA from tissues, tissues were first embedded in OCT compound and frozen sections were cut at 25 μm thickness and mounted on poly-l-lysin–coated LCM transfer film (LEICA-BEST) on glass slides. The chondrocytes in the tissue sections were dissected with a Leica LMD7000 Laser Microdissection System. RNA of the harvested chondrocytes was extracted with an RNAqueous Micro Kit (Ambion). Total RNA from cultured cells was isolated using a GenElute Mammalian Total RNA Miniprep Kit (Sigma-Aldrich). Total RNA samples were reverse-transcribed using High-Capacity cDNA RT Kits (Applied Biosystems) according to the manufacturer’s instructions. PCR reactions were performed with SYBR Premix Ex Taq (Takara Bio) on an ABI Prism 7300 thermocycler (Applied Biosystems). Standard plasmids ranging from 10^2^ to 10^8^ copies per well were used to quantify the absolute number of transcripts of cDNA samples. The numbers of transcripts were normalized to the number of transcripts of a house-keeping gene (GAPDH) in the same sample.

The primers used to determine mouse gene expressions were:

mGAPDH 5′-AGGAGCGAGACCCCACTAAC-3′ and 5′-CGGAGATGATGACCCTTTTG-3′;

mLPA_1_ 5′-AGGAGGAATCGGGACACCA-3′ and 5′-AGCACACATCCAGCAATAACAA -3′;

mLPA_2_ 5′-TGCCGCTTGACTGGATGT-3′ and 5′-GCTCCTTGCCGCTGTTATT-3′; mLPA_3_ 5′-ACCAACGTCTTATCTCCACACAC-3′ and 5′-CAGCAGCAGAACCACCAGAC-3′;

mATX 5′-GGAGAATCACACTGGGTAGATGATG-3′ and 5′-ACGGAGGGCGGACAAAC-3′;

mFibronectin 5′-CAGAACCAGAGGAGGCACAAG-3′ and 5′-CAATGGCGTAATGGGAAACC-3′;

mCollagen type II 5′-AGAGGGGACTGAAGGGACAC-3′ and 5′-GCACCCTGATCTCCAGAAGG-3′.

### Evaluation of Fibronectin assembly

Cells were plated on Col II-coated glass coverslips and stimulated with LPA after 24 hr starvation for 24 hr. To measure FN incorporation, 1 μg/ml Hilyte-488 FN (Cytoskeleton) was added with LPA. After 12 hours, cells were fixed and stained. Cells were treated with Ki16425 (5 μM), Y27632 (10 μM), GRGDSP (500 μM) or GRGESP (500 μM) for 30 min before LPA stimulation. PTX (200 ng/mL) treatment was applied at the same time as starvation. Fluorescent images were captured with the LSM700 confocal microscope equipped with 63×/1.40 Oil DIC M27 Plan-Apochromat.

### Decellularization

Cells were plated in 24-well plates with glass coverslips or 96-well plates at 1 × 10^5^ cells per well and cultured in the growth medium. After 10 days, cells were washed with PBS and treated with 0.5% Triton X-100 and 20 mM ammonium hydroxide in PBS for 5 min. Decellularized ECMs were treated with DNase (Sigma-Aldrich) at 50 unit/mL for 60 min at 37 °C, then gently washed with PBS. 24-well plates were stained with anti-FN antibody and images were captured with a Leica TCS SP-8 confocal microscope. 96-well plates were stored at −80 °C until use. To assay cell proliferation, cells were plated onto decellularized ECM plates at 0.5 × 10^4^ cells per well.

### Western blot analysis of deoxycholate (DOC)-insoluble fibronectin

After 10 days culture, cells were washed with PBS and solubilized with deoxycholate lysis buffer (DOC-buffer) containing 2% sodium deoxycholate, protease inhibitors (Complete Protease Inhibitor Cocktail, Roche), 20 mM Tris-HCl pH 8.8, 2 mM EDTA, 2 mM iodoacetamide and 2 mM N-ethylmaleimide. Homogenates were passed ten times through a 23-gauge needle, and centrifuged at 15,000 × g for 15 min at 4 °C. DOC-insoluble fractions were washed with DOC-buffer 3 times and then solubilized with SDS lysis buffer (1% SDS, 25 mM Tris-HCl pH 8.0, 2 mM EDTA, protease inhibitors, 2 mM iodoacetamide and 2 mM N-ethylmaleimide). Equal volumes of DOC-insoluble samples were analyzed by SDS-PAGE using 5% polyacrylamide gels. The primary antibody was goat anti-FN (Santa Cruz Biotechnology, sc-6952, 1:200). A secondary antibody conjugated with horse radish peroxidase (Dako, P0449, 1:1000) against goat IgG was used. Images were captured with a digital gel imaging system (ChemiDoc XRS^+^, BIO-RAD).

### Evaluation of ECM amount

After decellularization, ECMs were solubilized with 5.0 M Urea, 2.0 M Thiourea, 50 mM DTT and 0.1% SDS in PBS and scraped with a rubber policeman^63^. The collected lysates were placed in 95 °C water for 5 min and centrifuged at 12000 × g for 10 min at 4 °C. Protein concentration was evaluated by the Bradford method.

### Transmission electron microscopy (TEM)

Samples were fixed with 2% paraformaldehyde and 2% glutaraldehyde in 0.1M cacodylate buffer pH 7.4 at 4 °C overnight, washed 3 times with 0.1 M cacodylate buffer for 30 min each, postfixed with 2% osmium tetroxide in 0.1M cacodylate buffer at 4 °C for 3hr, dehydrated in graded ethanol solutions (50%, 70%, 90% and 100%), infiltrated with propylene oxide (PO) 2 times for 30 min each, transferred to a 70:30 mixture of PO and resin (Quetol-812, Nisshin EM Co.) for 1h, allowed to stand overnight to volatize the PO, transferred to fresh 100% resin and heated at 60 °C for 48 hr to polymerize the resin. Seventy-nm sections were cut from the polymerized resins (Ultracut UCT, Leica), mounted on copper grids, stained with 2% uranyl acetate at room temperature for 15 min, washed with distilled water, secondary-stained with lead stain solution (Sigma-Aldrich) at room temperature for 3 min and observed with a transmission electron microscope (JEM-1400Plus, JEOL) at an acceleration voltage of 80 kV.

### *In situ* hybridization

Heads from newborn mice were embedded in OCT compound. Seven-μm-thick sections were cut, placed on MAS-coated glass slides (Matsunami Glass), fixed with 4% PFA-PBS, acetylated with 0.5% (v/v) acetic anhydride/0.1M triethanolamine pH 8.0, permeabilized with 0.3% (v/v) TritonX-100/PBS, hybridized with digoxigenin labeled-RNA probes corresponding to 4551-4988 nt of Col2a1 cDNA (NM031163) or 1302-1816 nt of Col10a1 cDNA (NM009925), incubated with anti-digoxigenin antibody conjugated with alkaline phosphatase (Roche), stained with NBT/BCIP (BM purple AP, Roche Diagnostics) and photographed with a Zeiss Axio Imager (Carl Zeiss MicroImaging).

### Determination of lysophospholipase D activity

Lysophospholipase D activity of mice plasma was determined as described using 14:0 lysophosphatidylcholine as substrate^8^.

### Statistics

All statistical analyses were carried out using EXSAS. Differences were considered significant at *P* < 0.05. All data are expressed as means ± s.d.

## Additional Information

**How to cite this article**: Nishioka, T. *et al*. ATX-LPA_1_ axis contributes to proliferation of chondrocytes by regulating fibronectin assembly leading to proper cartilage formation. *Sci. Rep.* **6**, 23433; doi: 10.1038/srep23433 (2016).

## Supplementary Material

Supplementary Information

Supplementary Video 1

Supplementary Video 2

Supplementary Video 3

Supplementary Video 4
