# Supplementary material for: ATX-LPA1 axis contributes to proliferation of chondrocytes by regulating fibronectin assembly leading to proper cartilage formation
Source: Sci Rep. 2016 Mar 23;6:23433. doi: 10.1038/srep23433 (PMC4804234; doi:10.1038/srep23433)
Supplement: Supplementary Information [file srep23433-s1.pdf]

Scientific Reports

Supplemental Information

ATX-LPA$_1$ axis contributes to proliferation of chondrocytes by regulating fibronectin assembly leading to proper cartilage formation

Tatsuji Nishioka, Naoaki Arima, Kuniyuki Kano, Kotaro Hama, Eriko Itai, Hiroshi Yukiura, Ryoji Kise, Asuka Inoue, Seok-Hyung Kim, Lilianna Solnica-Krezel, Wouter H. Moolenaar , Jerold Chun and Junken Aoki

Supplemental material

Figure S1. Loss of ATX-LPA_1 signaling resulted in dyschondroplasia in zebrafish

(a) DNA sequence of wt and *lpa_1* mutant zebrafish induced by tilling assay. The gene structure of zebrafish *lpa_1* from exon1 – 3 is also shown. (b) Cartilages of zebrafish embryo around 96 hpf visualized by alcian blue and alizarin red staining (dorsal view). Schematic diagram is also shown. (c, d) Loss of LPA_1 signaling leads to deformation of gill cartilages. Length of Meckel's and ceratohyal cartilages in wt and *lpa_1* mutant zebrafish (c) and in vehicle- or Ki16425-treated wt zebrafish (d) (Data are mean ± s.d., n = 20-22, $^{**}P<0.01$, $^{***}P<0.001$). (e, f) Loss of ATX-LPA_1 signaling leads to deformation of cephalic region. Percentage of LPA_1 or ATX morphant embryos (e) and Ki16425-treated wt zebrafish embryos (f) with obvious cephalic malformation (Fig. 1b) is shown. (g) The expression patterns of *slug* and *sox10*, markers of CNCCs, in wt, LPA_1 or ATX morphant embryos. Scale bar: 50 μm.

Figure S2. Loss of ATX-LPA_1 signaling resulted in dyschondroplasia in mice

(a) Schematic diagram of intersphenoid synchondrosis in skull bone of mice. Expression marker of resting and proliferating zone (Col II) and prehypertrophic and hypertrophic zone (Col X) of cartilages and direction of bone expansion are also indicated. (b, c) Loss of ATX- LPA_1 signaling leads dyschondroplasia in mice. LPA_1 KO and $ATX^{flox/-}$ mice showed early ossification in intersphenoid synchondrosis (b), and mislocalization of chondrocytes both in costa and femur (c). Scale bar: 10 μm. (d-g) Decreased plasma ATX level induced dyschondroplasia in mice. (d) Plasma ATX level of wt, ATX HT and $ATX^{flox/-}$ (Data are mean ± s.d., n = 4). (e) Loss of LPA_1 signaling has no effect on the expression of chondrocyte differentiation markers (Col II and Col X) as judged by in situ hybridization. Scale bar: 50 μm. (f) Gene expression of *atx*, *lpa1*, *lpa2* and *lpa3* in intersphenoid synchondrosis (Data are mean ± s.d., n = 3).

Figure S3. Inhibition of LPA_1 signaling results in elongation of doubling time in cultured chondrocytes

(a) Inhibition of LPA_1 signaling resulted in decreased cell size. $LPA_1^{+/-}$ chondrocytes (HT) and $LPA_1^{-/-}$ chondrocytes (KO) were cultured in medium containing 10% FCS and LPA_1 antagonist (Ki16425) or ATX inhibitor (ONO-8430506). Scale bar: 10 μm. (b, c) Loss of LPA_1 signaling resulted in elongation of doubling time but did not affect duration of M phase. (b) HT and KO chondrocytes were cultured in medium containing 10% FCS and LPA_1 antagonist (Ki16425) or ATX inhibitor (ONO-8430506) for 48 hr. The doubling time (b) and duration of M phase (c) were measured from time lapse images (NT:

non-treated, Data are mean ± s.d., n = 10, * $P < 0.05$, **$P < 0.01$).

Figure S4. LPA-induced S-phase entry of chondrocytes is integrin-dependent also on Col II-coated plates

(a) HT and KO chondrocytes were stimulated with LPA and evaluated the cell spreading area on Col II-coated plates 12 hr after the stimulation (NS: non-stimulated, Data are mean ± s.d., N.S.: not significant, **$P < 0.01$, $^{###}P < 0.001$). (b) Effects of LPA_1 signal inhibitors (LPA_1 antagonist (Ki16425), ROCK inhibitor (Y27632) or PTX) on LPA-induced S-phase entry on Col II-coated plates (NS: non-stimulated, Data are mean ± s.d., n = 4, ***$P < 0.001$, $^{##}P < 0.01$). (c) LPA-induced S-phase entry is integrin-dependent also on Col II-coated plates. LPA-induced S-phase entry of HT chondrocytes was evaluated by BrdU incorporation in the presence of integrin-blocking peptide (GRGDSP) and control peptide (GRGESP) (NS: non-stimulated, Data are mean ± s.d., n = 4, N.S.: not significant, ***$P < 0.001$, $^{###}P < 0.001$).

Figure S5. LPA promotes formation of focal adhesions through LPA_1

LPA promotes formation of focal adhesions through LPA_1, $G_{\alpha 12/13}$, $G_{\alpha i}$ and integrin-mediated signaling. Chondrocytes were stimulated with LPA in the presence or absence of LPA_1 antagonist (Ki16425), ROCK inhibitor (Y27632), PTX ($G_{\alpha i}$ inhibitor). Cells were immunostained with anti-vinculin, anti-β1-integrin antibody and phalloidin. Scale bar: 5 μm.

Figure S6. Decellularization of cultured chondrocytes

(a) The cell number of HT and KO chondrocytes cultured in medium containing 10% FCS at high density for 10 days. (b) Transmission electron micrographs of ECM formed by HT or KO chondrocytes cultured in medium containing 10% FCS at day10. Scale bar: 5 μm and 200 nm in magnified view. (c) Chondrocytes were cultured for 10 days. To perform decellularization, the cells were treated with Triton X-100, ammonium hydroxide and DNase. Then the plates were immunostained with anti-FN antibody and phalloidin. Scale bar: 10 μm.

Video 1

Time lapse images of $LPA_1^{+/-}$ chondrocytes cultured in medium with 10% FCS for 48 hr.

Video 2

Time lapse images of $LPA_1^{-/-}$ chondrocytes cultured in medium with 10% FCS for 48 hr.

Video 3

Time lapse images of $LPA_1^{+/-}$ chondrocytes cultured in medium with 10% FCS in the presence of LPA_1 antagonist (Ki16425) for 48 hr.

Video 4

Time lapse images of $LPA_1^{+/-}$ chondrocytes cultured in medium with 10% FCS in the presence of ATX inhibitor (ONO-8430506) for 48 hr.

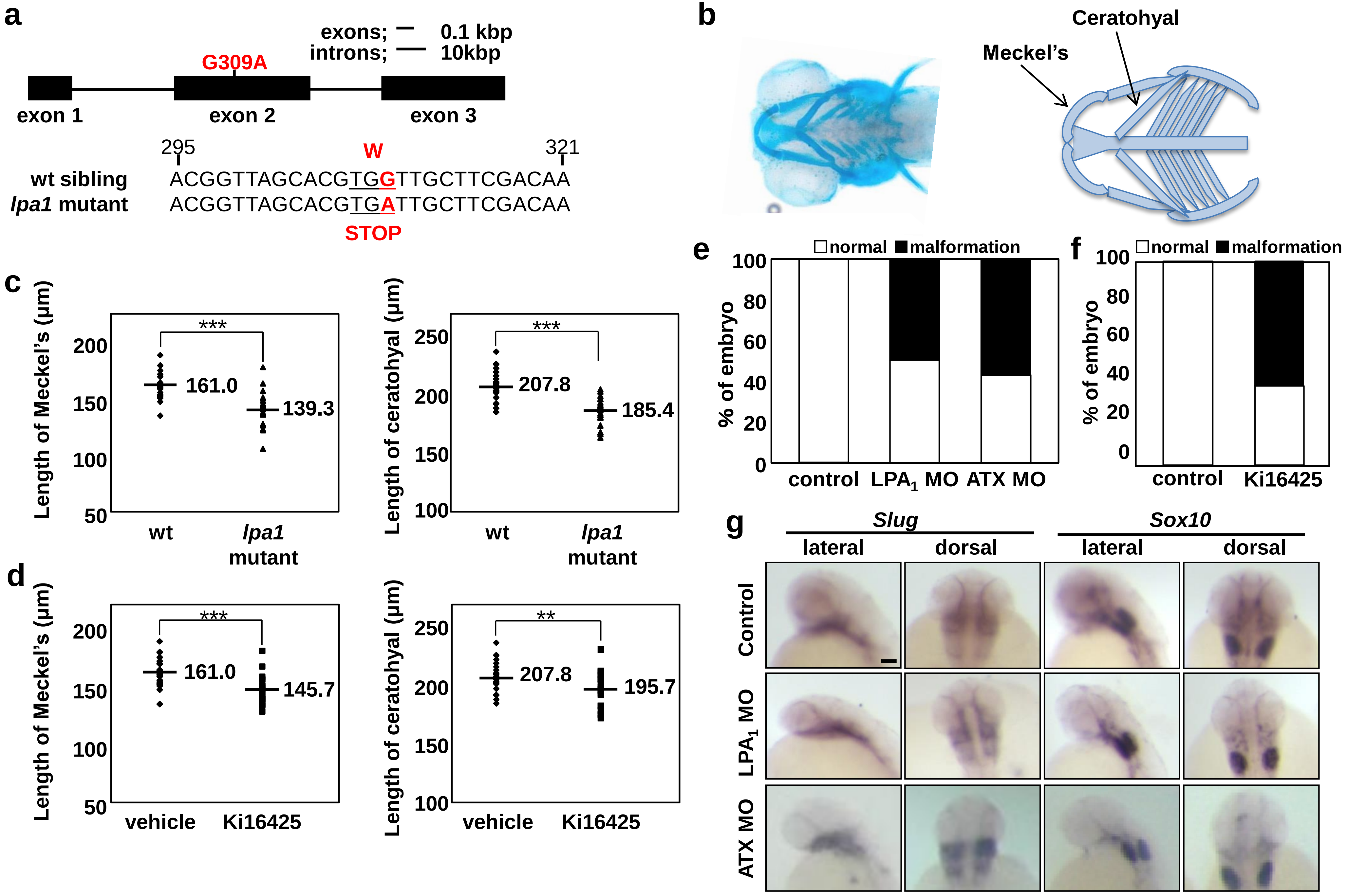

a
exons; 0.1 kbp
introns; 10kbp
G309A
exon 1
exon 2
exon 3
295
W
321
wt sibling ACGGTTAGCACGTGGTTGCTTCGACAA
lpa1 mutant ACGGTTAGCACGTGATTGCTTCGACAA
STOP
b
Ceratohyal
Meckel's
c
Length of Meckel's (μm)
161.0
139.3
wt
lpa1 mutant
Length of ceratohyal (μm)
207.8
185.4
d
161.0
145.7
vehicle
Ki16425
207.8
195.7
e
normal
malformation
% of embryo
control
LPA1 MO
ATX MO
f
control
Ki16425
g
Slug
Sox10
lateral
dorsal
Control
LPA1 MO
ATX MO

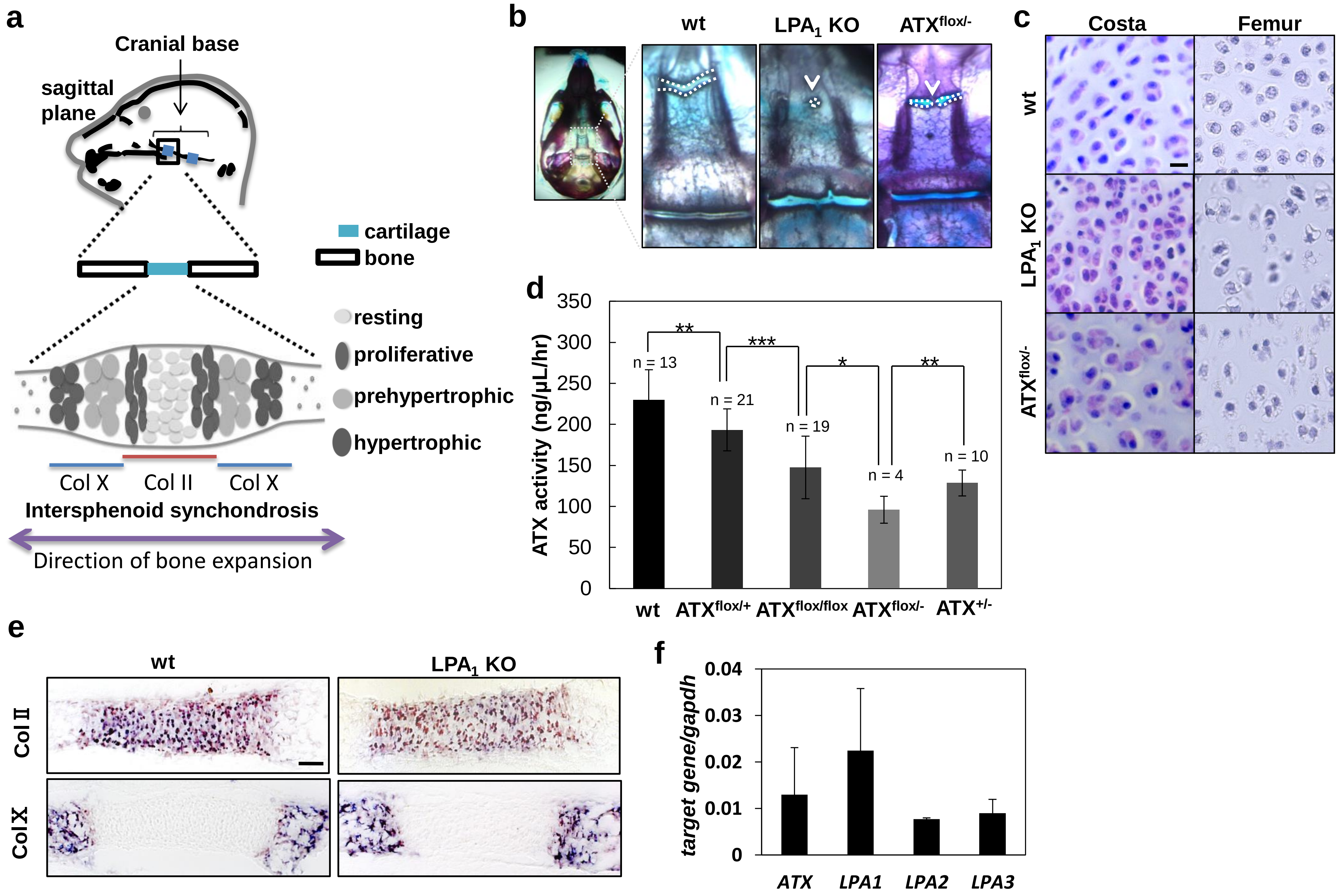

a
Cranial base
sagittal plane
cartilage
bone
resting
proliferative
prehypertrophic
hypertrophic
Col X
Col II
Col X
Interspheniod synchondrosis
Direction of bone expansion
b
wt
LPA1 KO
ATXflox/-
c
Costa
Femur
wt
LPA1 KO
ATXflox/-
d
ATX activity (ng/μL/hr)
350
300
250
200
150
100
50
0
**
***
*
**
n = 13
n = 21
n = 19
n = 4
n = 10
wt
ATXflox/+
ATXflox/flox
ATXflox/-
ATX+/-
e
wt
LPA1 KO
Col II
Col X
f
target gene/gapdh
0.04
0.03
0.02
0.01
0
ATX
LPA1
LPA2
LPA3

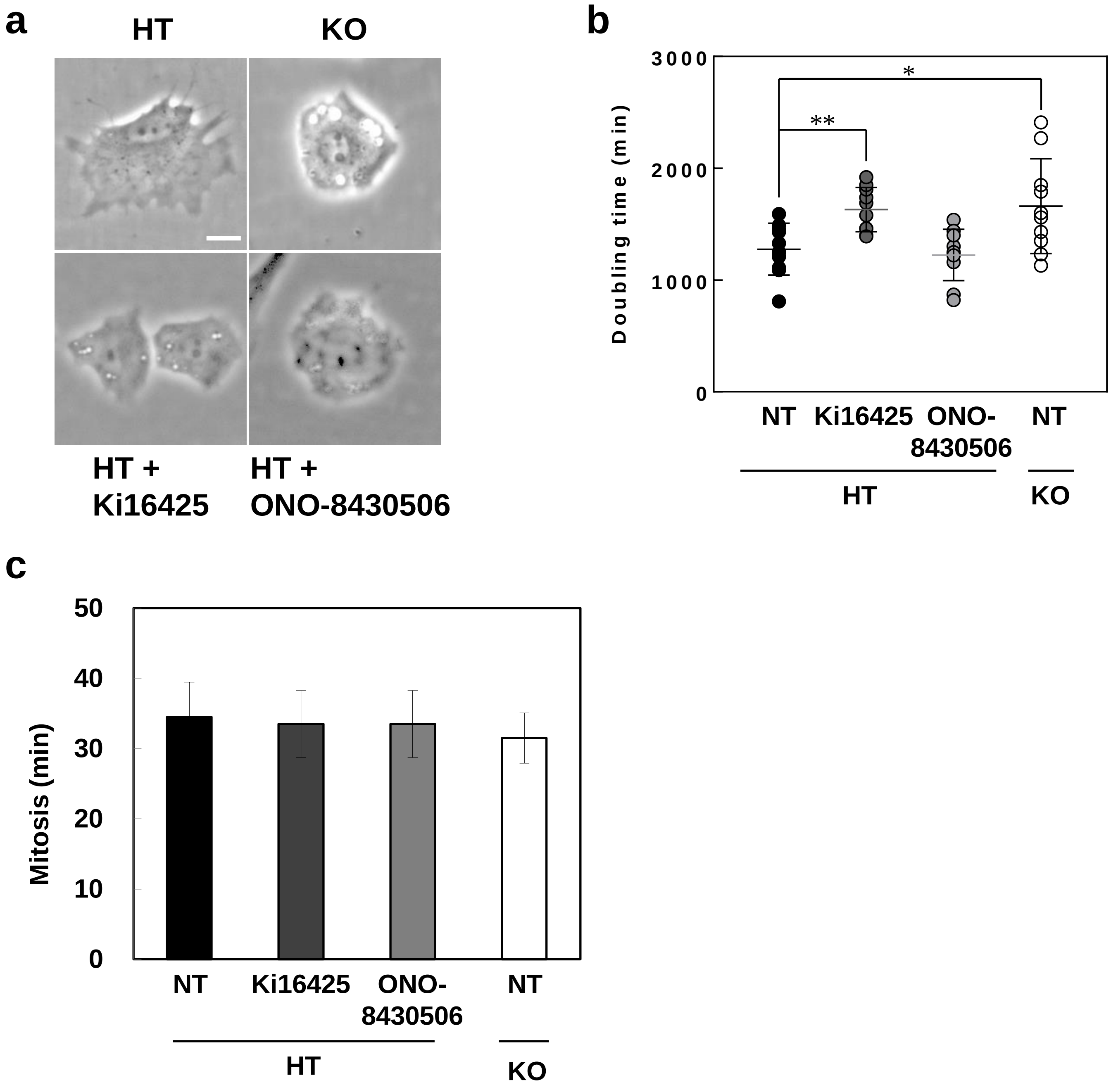
a
HT
KO
HT + Ki16425
HT + ONO-8430506
b
Doubling time (min)
3000
2000
1000
0
*
**
NT
Ki16425
ONO-8430506
NT
HT
KO
c
Mitosis (min)
50
40
30
20
10
0
NT
Ki16425
ONO-8430506
NT
HT
KO

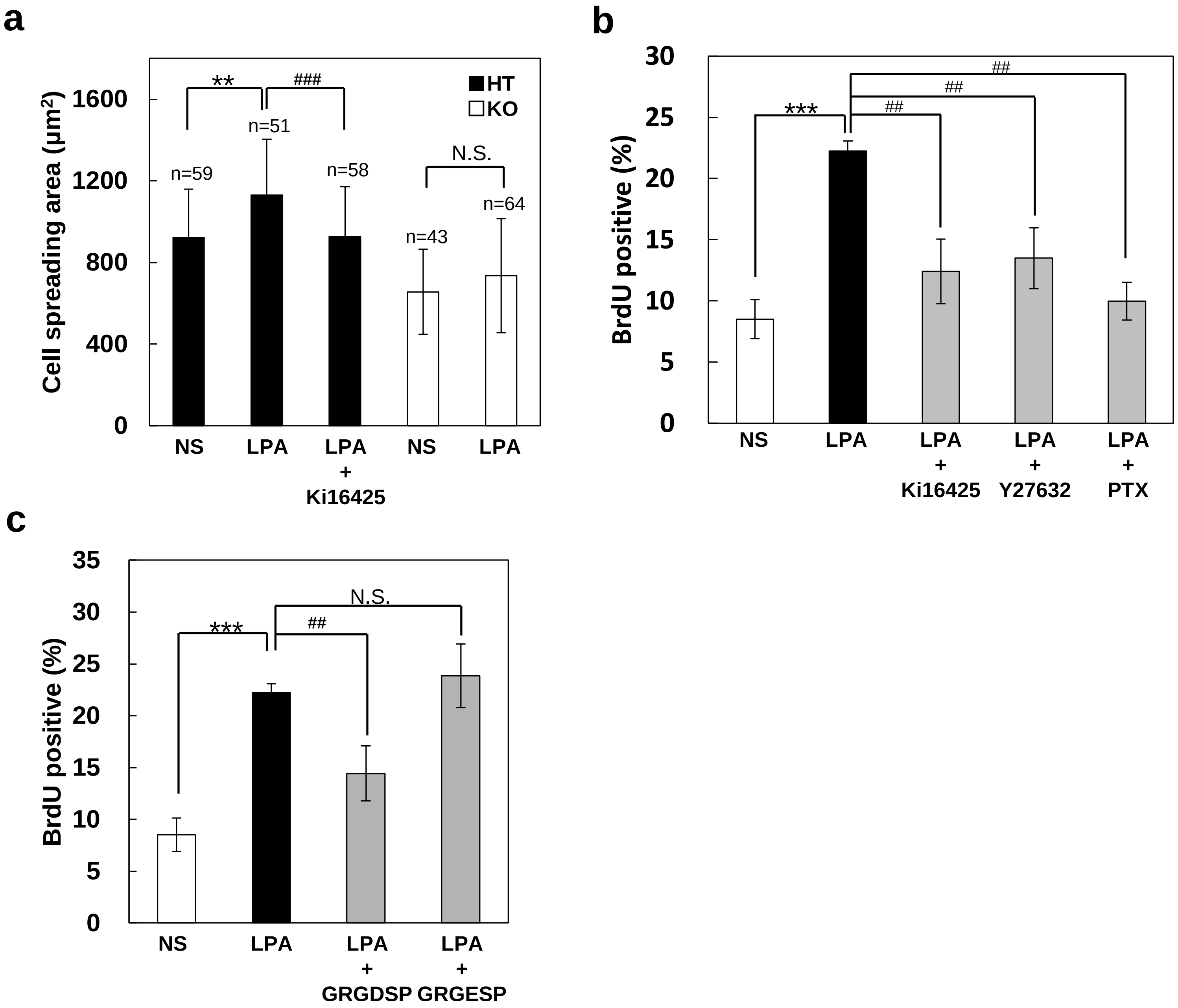
a
Cell spreading area (μm²)
0
400
800
1200
1600
■HT
□KO
**
###
N.S.
n=59
n=51
n=58
n=43
n=64
NS
LPA
LPA + Ki16425
NS
LPA
b
BrdU positive (%)
0
5
10
15
20
25
30
***
##
##
##
NS
LPA
LPA + Ki16425
LPA + Y27632
LPA + PTX
c
BrdU positive (%)
0
5
10
15
20
25
30
35
***
##
N.S.
NS
LPA
LPA + GRGDSP
LPA + GRGESP

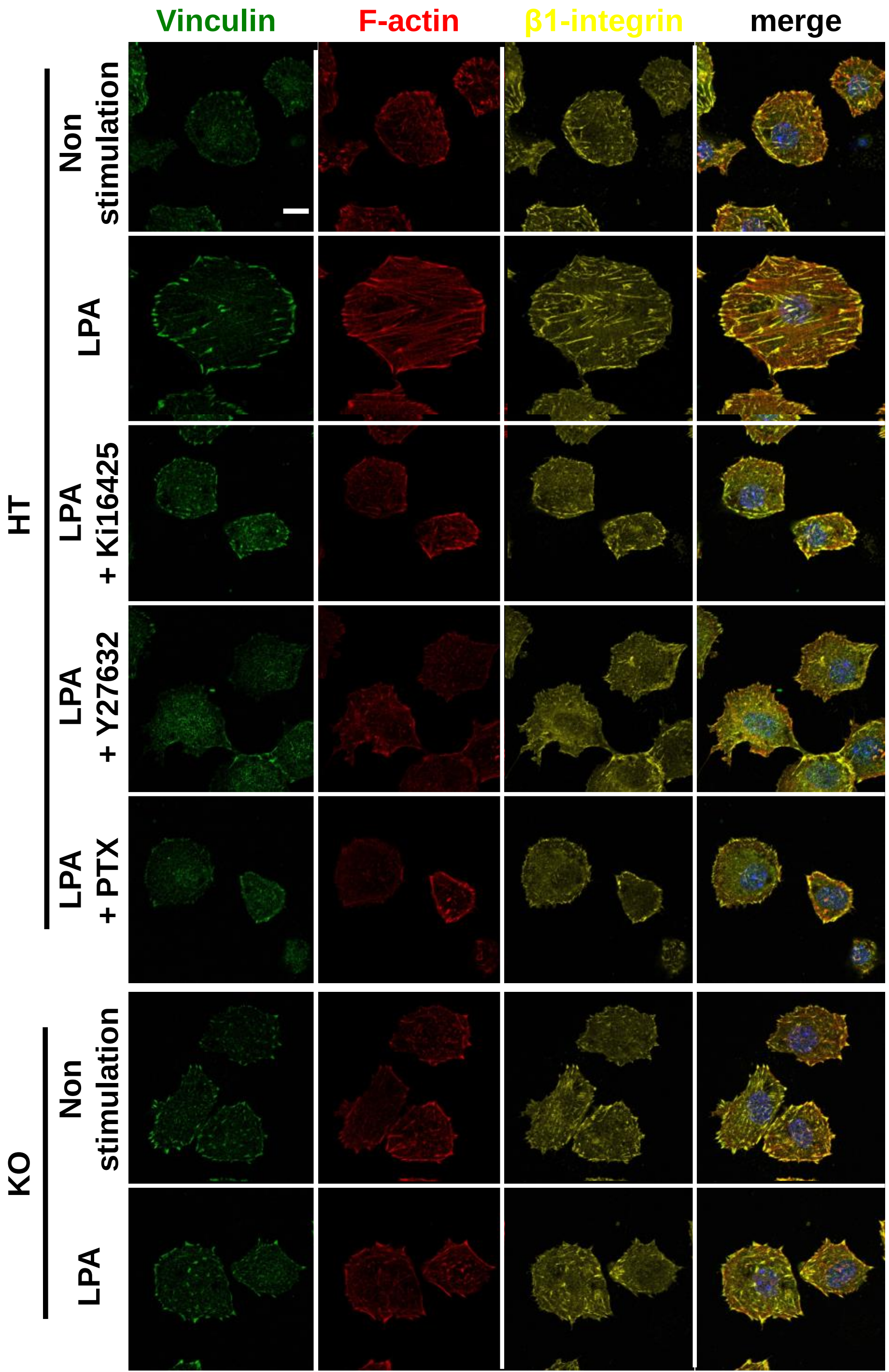

Vinculin
F-actin
β1-integrin
merge
HT
Non stimulation
LPA
LPA + Ki16425
LPA + Y27632
LPA + PTX
KO
Non stimulation
LPA

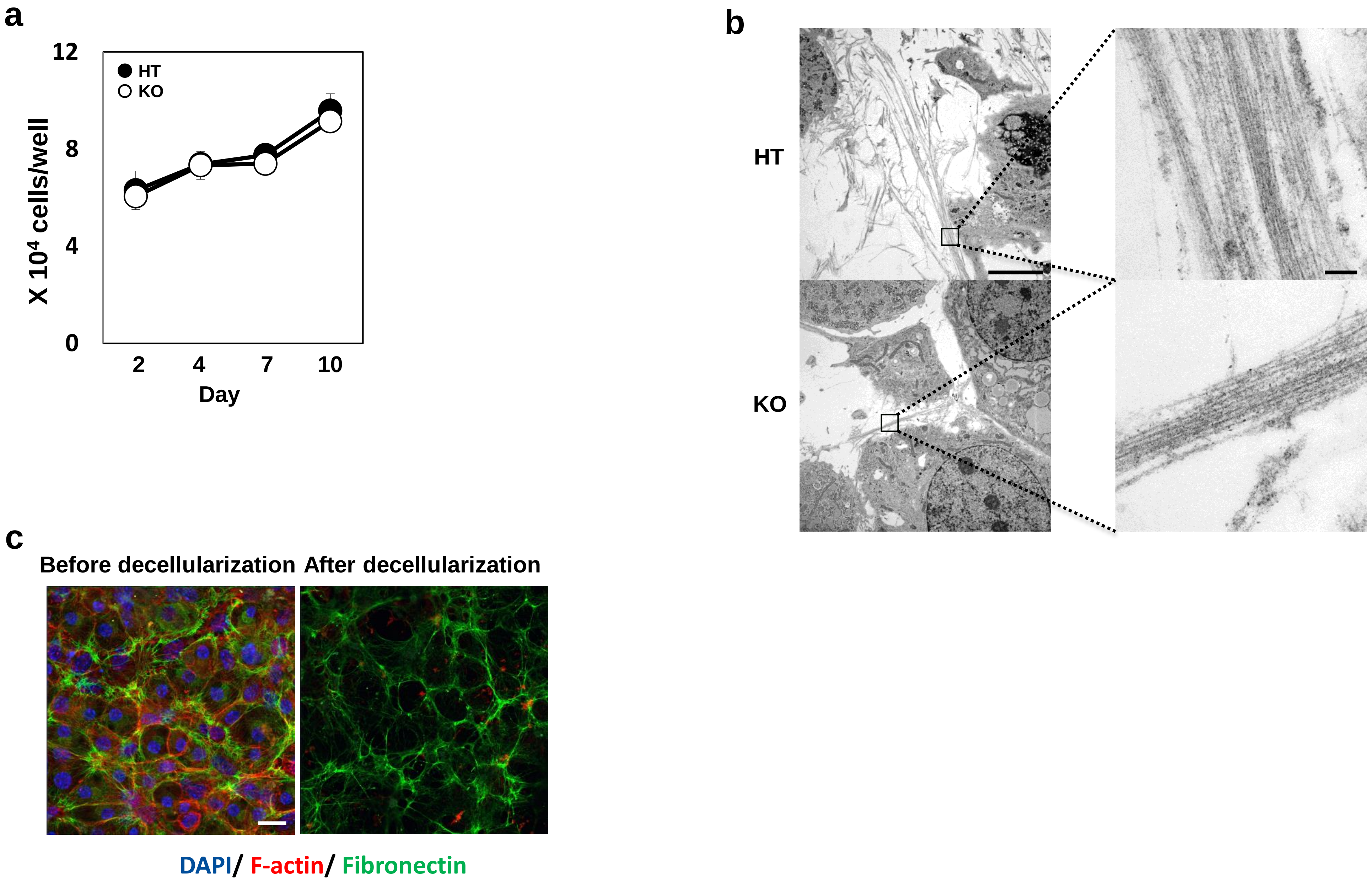
a
12
8
4
0
X 10^4 cells/well
HT
KO
2
4
7
10
Day
b
HT
KO
c
Before decellularization
After decellularization
DAPI/ F-actin/ Fibronectin
